# Hinge craniotomy as an alternative technique for patients with refractory intracranial hypertension

**DOI:** 10.1016/j.bas.2023.101758

**Published:** 2023-05-19

**Authors:** Ibrahim Omerhodzic, Almir Dzurlic, Bekir Rovcanin, Kresimir Rotim, Amel Hadzimehmedagic, Adi Ahmetspahic, Zlatan Zvizdic, Nermir Granov, Enra Suljic

**Affiliations:** a Department of Neurosurgery, Clinical Center University of Sarajevo, Sarajevo, Bosnia and Herzegovina; b Department of Neurosurgery, Clinical Hospital Center Sisters of Mercy, Zagreb, Croatia; c Department of Cardiovascular Surgery, Clinical Center University of Sarajevo, Sarajevo, Bosnia and Herzegovina; d Department of Pediatric Surgery, Clinical Center University of Sarajevo, Sarajevo, Bosnia and Herzegovina; e Department of Neurology, Clinical Center University of Sarajevo, Sarajevo, Bosnia and Herzegovina

**Keywords:** Decompressive craniectomy, Hinge craniotomy, Refractory intracranial hypertension, TBI, ICP

## Abstract

**Introduction:**

Decompressive craniectomy (DC) can save brain tissue, but unfortunately it has many limitations and complications. Hinge craniotomy (HC), as less aggressive method seems to be adequate alternative not only to DC but also to conservative treatment.

**Research question:**

Presentation of the results of modified surgical techniques of cranial decompression and comparing with more and less aggressive medical options.

**Material and methods:**

A prospective clinical study was conducted during 86 months. Comatose patients who suffered refractory intracranial hypertension (RIH) were treated. Altogether, 137 patients have been evaluated. The final outcome of all patients in the study was evaluated after 6 months.

**Results:**

Both surgical options resulted in adequate control of intracranial pressure (ICP). HC method was shown to have the lowest probability of worsening from a prior state of relative stability.

**Discussion and conclusion:**

There was no statistically significant difference between methods to treatment of DC or HC, meaning the final outcome of patients treated in any manner. There was similar rate of early and late complications.

## Highlights

• No statistically significant difference exists in regards to efficacy between by DC, HC and conservative treatment.
• Patients with temporal contusions of the brain, unilateral edema, massive MCA infarction and acute subdural hematoma, were shown to have larger benefit from HC.
• Early surgical intervention, better GCS on admission, and relatively younger patients showed better outcomes after decompression.
• Patients with refractory intracranial hypertension after severe TBI, DAI and aSAH are likely to be better treated with DC.

## 1 Introduction

Injuries and damage of the highly sophisticated tissue of the brain has engaged thousands of researchers, primarily due to the human emotional desire to help those in need, but also as a result of the social, societal and economic aspects and consequences of such traumatic events.

Traumatic Brain Injury (TBI) leads to early and/or chronic cognitive and physical disability with incidence rates ranged from 47.3 to 694/100,000 population per year (Brazinova et al., 2021). Increased intracranial pressure (ICP) is one of the leading causes of death and disability after a severe TBI and stroke (Carney et al., 2017). Increased ICP is a very serious medical condition, because the skull cannot widen, and thus the soft, delicate and sophisticated brain tissue in these events becomes compressed and comprised within the closed intracranial “cage”. As a result, a complete alteration of neural function ensues, and if this emergency continues and is not treated quickly, the high ICP leads to permanent morbidity and mortality (Donnelly et al., 2020). Thus, it is imperative for patients with increased ICP to be treated acutely in the best possible manner.

Intracranial hypertension (IH) is mostly defined by episodes of ICP >20 ​mmHg, which lasts for more than five to 10 ​min, and usually requires medical intervention, while refractory intracranial hypertension (RIH) is typically defined as an ICP >25 ​mmHg and is a life-threatening situation (Carney et al., 2017; Donnelly et al., 2020). This state requires remarkably aggressive methods of treatment as a final option, such as barbiturate induced coma or decompressive craniectomy (DC). (Kolias et al., 2018).

An acute increase in ICP, which returns to normal levels within 5 ​min, is not considered as significant; however, if levels remain above 20 ​mmHg for prolonged states, these conditions may require additional surgical intervention and therapy. RIH may be characterized as a failure of standard therapy without decompression (first line) for control of ICP (Stocchetti and Maas, 2014; Hutchinson et al., 2013). This situation is found in about 10–15% of cases. Refraction may point to a poor diagnosis, with mortality larger than 80% in the case of RIH. In this situation, it was essential to utilize second line therapeutic treatment options, which are characterized by their complexity and potentially fatal adverse effects (Omerhodzic et al., 2014).

Decompressive craniectomy is a surgical procedure during which part of the skull, usually directly above the damaged region of the brain, is temporarily removed, which enables additional room for swelling of the brain and thus decreased ICP, and therefore prevents direct mechanical damage of the brain (Badri et al., 2012). More specifically, this procedure saves brain tissue locally in the region of surgical intervention, but also indirectly, in more distant brain structures (Badri et al., 2012; Timofeev et al., 2012). Reaching the decision to intervene by a decompressive procedure alone in comparison to more conservative treatment, *a priori*, is not sufficient (Omerhodzic et al., 2014; Cooper et al., 2011a). The impacts of DC, from the neurosurgical perspective, towards the stances which have been upheld until the start of our study, have been insufficiently confirmed at best (Hutchinson et al., 2006a).

Limited referrals upon at most several smaller retrospective trials, which included a small number of patients with TBI and stroke, showed that, in some patients, hinge craniotomy (HC) may be a useful procedure in the treatment of acute edema of the brain, IH or RIH (Kenning et al., 2012; Kano et al., 2012). Hinge craniotomy is a modification of DC, where is the bone flap preserved *in situ* in a ‘floating’ or ‘hinged’ fashion, as well as protection of the brain from new, external injuries, and avoided is the needed for reimplantation of bone later on. Hinge craniotomy is reported to be good as DC in controlling of ICP in TBI/stroke patients, but there is some evidence of reduction of complication and infection rates in HC compared with DC, but there is no enough evidence, and comparation of HC vs. DC (Kenning et al., 2009; Yang et al., 2008a).

The results of the aforementioned studies showed that the aggressive DC does not necessarily result in better outcomes than more conservative medical treatment. Carrying the information above in mind, hinge craniotomy is a procedure which is imposing itself as a potential separate operative technique, useful in solving problems associated with DC, which are commonly listed in available literature.

## 2 Goal

Standardization of one of the methods of decompression for specific groups or subgroups of selected patients. Presentation of relatively new, modified, surgical techniques of cranial decompression, with the accent on their controlled and objectified evaluation of utility.

## 3 Patients and methods

In this research a prospective clinical study was conducted. The research was begun with permission of all relevant institutions and establishments, and was finalized with the results of treatment and outcomes of neurosurgical patients with increased ICP, refractory to standard medically conservative therapy. The main interest was focused on the application of HC, as an elegant surgical technique compared to the typical DC technique, for the treatment of RIH. RIH was attributed to those cases of intracranial hypertension which had values of over 20 ​mmHg, but which lasted for 15 ​min continuously or for several episodes whose collective sum was 15 ​min or longer, within 1 ​h, after previous treatment with hyperosmolar therapy, head elevation and sedation. The study encompassed 137 patients, who during the study were consecutively admitted or were treated in the Neurosurgical Intensive Care Unit (NICU). Preoperatively, according to the usual procedure, valid consent for operations and interventions was enabled, which also included procedures from the study.

In the period of 86 months, patients were treated, and followed, with ranges 18 to 65, with varying pathology which led to increased ICP and clearly worsening neurological states, and also typically a direct risk to patient livelihood. Patients received the best methods of treatment which were available at the time, according to widely accepted guidelines. The primary outcome was graded using the Glasgow Outcome Scale (GOS) during two time periods: GOS0, at the moment of departure from the neurointensive care unit, and at GOS6, a time-point six months following the hospital admission. Special attention was paid to the timeline of disease for those being treated with HC or DC due to increased or refractory ICP, who were initially placed in a NICU. During the admission, generally, all included patients were comatose (GCS 3-8).

**Fig. 1:**
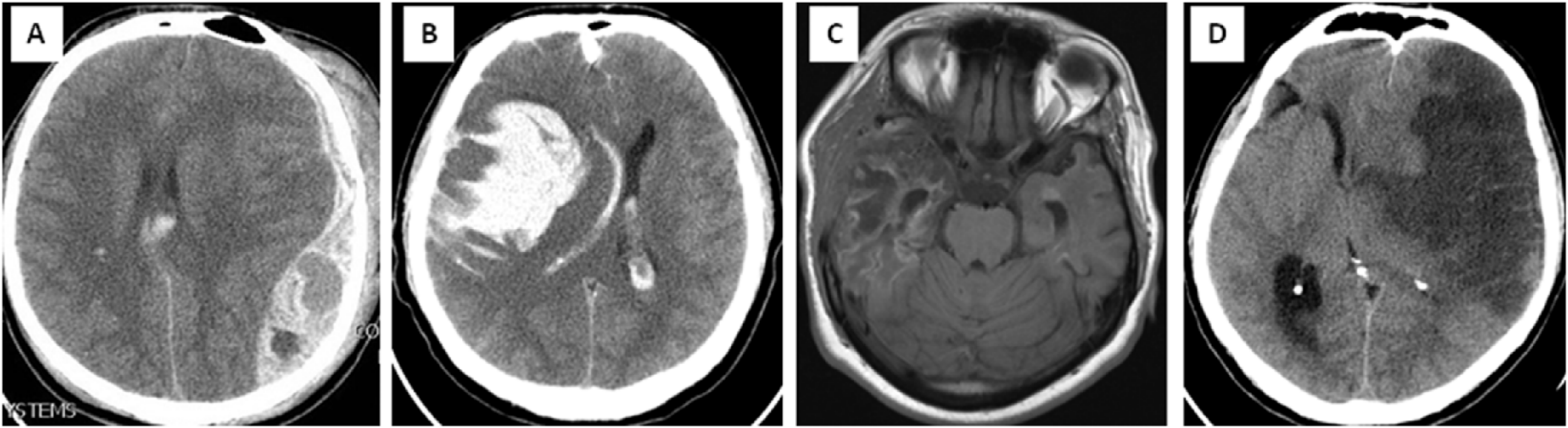
Scans of four representative patients from the study. Patients were treated for a different pathology which caused intracranial hypertension. Three patients were operated on and survived, with residual sequelae, but able to perform daily functions independently. One patient was treated only conservatively, and died. A) Axial CT of a 22-year-old male, with sTBI and traumatic ultraacute ASDH on the left; brain edema and midline shift is visibile; at addmition was GCS 5; urgently treated surgically – hematoma was evacuated and primary DC was performed. B) Extensive intracerebral hematoma in a 37-year-old man with aSAH and edema on the right, caused by a ruptured cerebral aneurysm; at addmission was GCS 4; emergency surgery was done, hematoma evacuated, MCA aneurysm clipped, and Hinge craniotomy was performed. C) Young 26-year-old girl, complication of meningitis and local cerebritis on the left temporal region, followed with thrombosis of middle cerebral artery and stroke; after deterioration of consciousness to GCS 7 she was admitted to neurosurgical department from the infectious disease clinic, and urgently operated - secondary DC was perfomed; early follow up MRI of the brain showed a combination of local infection and insult, with brain fungus temporobasally, at the site of decompression. D) Middle-aged man, 47 years old, transferred from the neurological clinic to the NICU, due to consideration for decompression concerning deterioration of consciousness after malignant MCA stroke verification; GCS was 6; no surgery was done; patient died third day after onset of stroke. (sTBI - severe TBI; aSAH - aneurysmal subarachnoid hemorrhage; ASDH - acute subdural hematoma; MCA - middle cerebral artery)

All respondents were in essence those who possessed one of the following diagnoses: traumatic intracranial hemorrhage (either requiring evacuation of the hematoma or not); brain edema due to diffuse axonal injury; massive MCA or ICA infarct; aneurysmal SAH; hemorrhagic stroke; thrombosis of the venous sinus and/or magistral veins; infections disease of the brain; and diffuse edema of unknown etiology (Fig. 1).

**Fig. 2:**
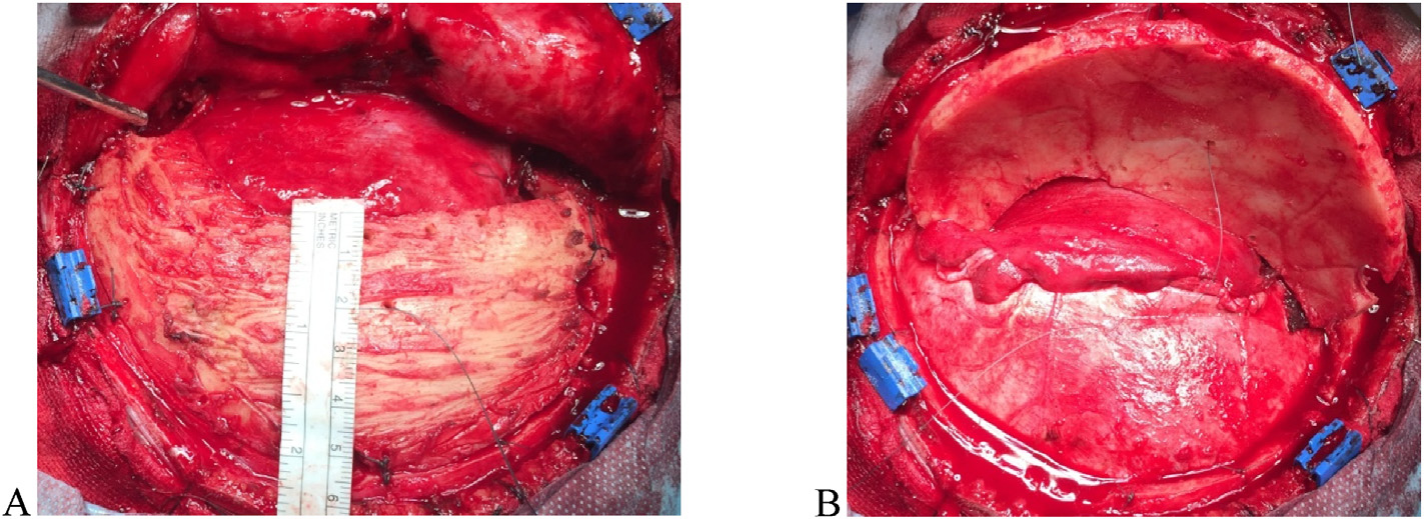
Presentation of Hinge craniotomy technique. A) Bone flap size ≥12 ​× ​6 cm, with extensive basal craniectomy, basal third of bone flap is removed, including lateral wall and part of floor of middle fossa, to better relieve of temporal lobe compression to brainstem; The remaining upper part of bone flap is wide enough to cover the defect and subsequent cranioplasty is not necessary; B) Dural closure and dural pocket are made with the fascia of temporal muscle, which allows the brain to “breathe” outwards due to edema and elevated ICP through a sufficiently wide Hinge window. Bone flap is elevated contrary to Hinge – to better understanding of decompressed dural exposure.

**Fig. 3:**
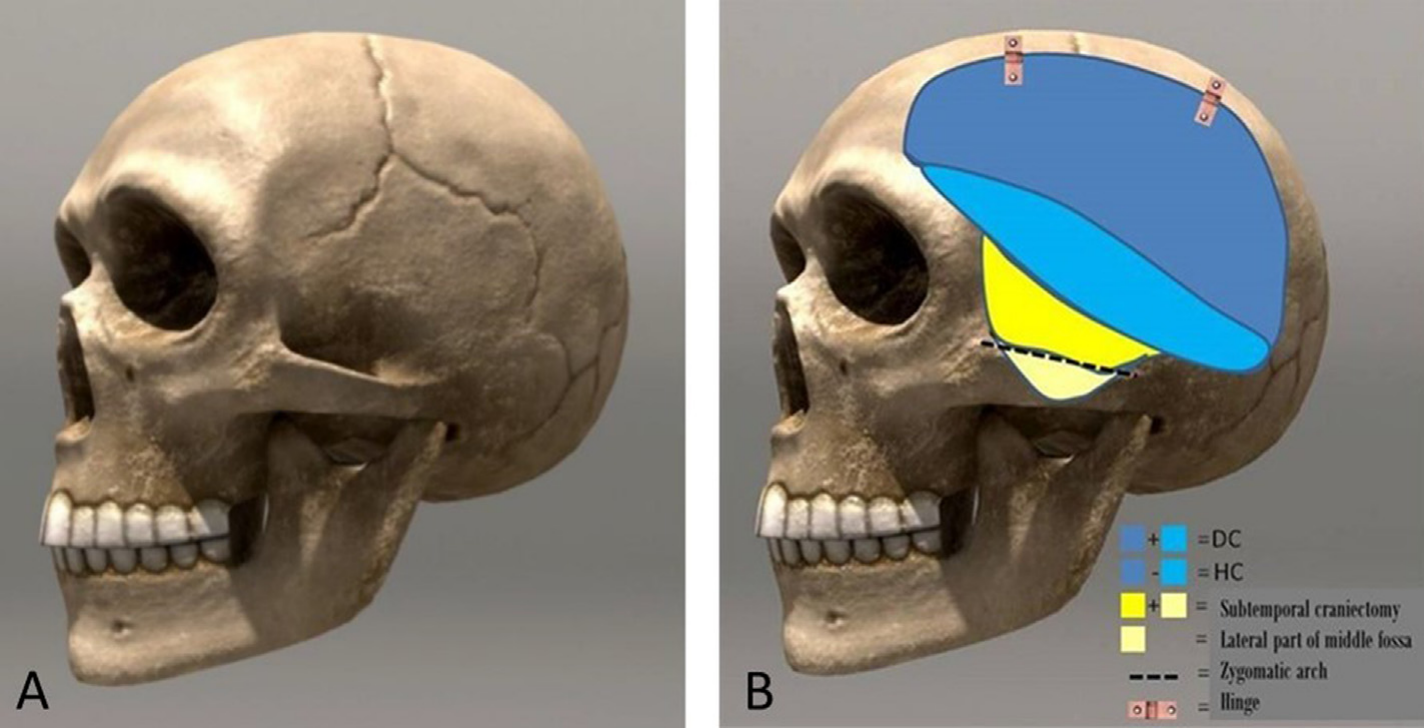
Comparison of classic DC with Hinge decompression. A) The landmark sutures are shown as important for determining the decompression area. B) Schematic adopted image shows the area traditionally removed with the DC technique (indicated in blue). Most lower part of the outer wall of the middle cranial fossa should also be removed (colored yellow). With a Hinge craniotomy, we emphasize this lower part of the flap. Additionally, we recommend removing the lateral part of the floor of the middle fossa (slanted part, marked in beige in the image). It is not necessary to remove part of the zygomatic arch. At the end, the upper two-thirds of the bone flap (dark blue) are returned to its place, and positioned loosely on metal hinges or simply with wires or sutures, allowing the movement of the bone flap inwards and outwards, depending on the requirements of the edematous or “quiet” brain. (For interpretation of the references to color in this figure legend, the reader is referred to the Web version of this article.)

Patients sampled were divided into three groups, according to treatment technique. Neurosurgeon who was treating the patient decided based on his expertise which treatment technique was used. All three groups had comparable prognoses and were treated in a standard fashion. The first tested group (DC group) was exposed to therapeutic procedures of decompressive craniectomy, per its indication. The second group (HC group) was, along with standard therapy exposed to surgical interventions of hinge craniotomy as well. The control group (K group) was not exposed to surgical procedures of decompression, but contained patients who were only conventionally medically treated, according to respective guidelines and indications (Fig. 2, Fig. 3). The patients in K group were treated in intensive care unit with conservative medical treatment, sedation, hypothermia and/or external ventricular drainage.

All three groups were intensively chronologically followed, and evidenced was the clinical picture of patients, whether the conducted therapy was conservative and/or operative, results of the postoperative rehabilitation treatment, and recorded follow-up outcomes. Recordings were conducted according to official forms and evidence notebooks, in printed form of the patient's medical history, discharge letter and operative records, as well as in digital form. For the study, conducts were specifically designed, all-encompassing written and digital protocols of events during the research. The results were evaluated quantitatively-qualitatively, according to internationally accepted scales, especially the Glasgow Coma Scale (GCS), Glasgow Outcome Score scale (GOS), Karnofsky scale, and other qualitative criteria and methods. In the computer database (Microsoft Office Excel) through data forms, logged was all relevant data which dealt with remission of disease, and so the possibility of primary and additional later comprehensive analysis was simplified.

### 3.1 Hinge surgical technique

Patients underwent a standard craniotomy in the operating room, aimed at the presented pathology. The difference between DC and HC is that with HC we leave the bone flap, which give as a little cosmetic defect and also there is no need for cranioplasty. After evacuation of the hemorrhagic lesion (if it was necessary), dura plastic is performed with artificial dura (liodura) or galeoma, which is sewn to the dural edges, and it can be combined, both dura and galea or fascia.

The lower (inferior, caudal) edge of the bone flap is attached with a mini straight titanium plate and screw. The screw is not turned all the way, but remains protruding (protrudes) above the edge of the bone by 1–2 ​mm, so that the bone flap is somewhat mobile in to that part. The titanium plate is fixed to the flap in the same, “loose” way. With this the procedure actually makes a “hinge” that only partially attaches the flap (in situ) for the cranium, but flap mobility is still significant. On the upper part of the flap (superior, cranial) plates are fixed only for the flap, and it is left complete the freedom of the flap to oscillate outwards or inwards, depending on intracranial tension and movement of the brain mass, according to the current pathophysiology of the brain.

The subgaleatic space is further enlarged by blunt dissection (“by undermining”) between the pericranium and the galea, thus facilitating excursion bone flap outwards, in case of brain expansion. Galea, further, can be incised (galeotomy) parallel to the scalp incision, which enables additional expansion of this space. If the curved bone flap is thick, the internal lamina can be removed and thin the flap. Finally, if it has been removed, it can be posted again ventricular catheter, preferably 2–3 ​cm beyond the edge of the craniotomy, for ICP monitoring.

## 4 Results

In total the study included 137 patients. The first patient was registered in the study in June 2011 and the last patient who was included was admitted into the hospital at the beginning of June 2018. During the study the DC group claimed 43 patients, the HC group 45, and the K group 49. There were 77 males, more specifically according to their representation among groups: DC 24, HC 21, and K group had 32, while on the other hand, there were 60 women, and according to their representation among the three groups, in the DC there were 19, in the HC 24, and in the K 17. Of the 88 patients treated with decompression, 57 patients were primarily evaluated for decompression while 31 were approved for and operated later on due to their worsening clinical picture. At the end of the study, patient outcomes from all three groups (DC, HC, and K) were mutually compared, in the same time periods. We evaluated which methods produced the best results, and we also reviewed whether we adequately decided upon either operative and/or conservative methods of treatment, and could have we acted better therapeutically.

The final outcome of all patients in the study was evaluated after six months. The average age of patients in the groups was uniform, that is, 48.7, 43.0, and 49.3 years for DC, HC and the K groups respectively. A similar number of patients in all three groups received analogous and comparable medical treatment. There were no significant differences between the groups in the prevalence of craniotomies conducted due to evacuation of hematomas between groups DC and HC. A total of 112 (81.7%) of patients were sedated prior to the operation (85% in the DC and 77% in the HC group). Decompressive craniectomy was conducted in 31.1% of patients, while Hinge was conducted in 32.8%, the need for cranioplasty in survivors was significantly higher in of patients who underwent decompressive craniectomy (100%) of those who were treated with Hinge craniotomy (12%).

In the essential demographic, neurological status, CT imaging characteristics (according to the Marshall score) and clinical picture (graded on the basis of the Rotterdam score) there were no significant differences in results between patients treated with HC and those treated with the DC technique. Table 1.

**Table 1:** Comparison of groups according to age, condition at admission and fitness.

Parameter	DC Group (n 43)	HC Group (n 45)	K Group (n 49)	*p*
Age	48,7	43,0	49,3	NS
GCS on admission	5,8	5,9	5,8	NS
Diffuse TBI	12	11	9	NS
TBI with hematoma/contusion evacuation	27	27	10	NS
Ischemic stroke	1	3	11	NS
Vasospasm in aneurysmal SAH	2	3	14	NS
Infection, non-traumatic mass lesion, etc.	1	1	5	NS
Closed basal cisterns	39	40	38	NS

(n, number of patients; p, level of significance; NS, no significant difference).

**Chart 1:**
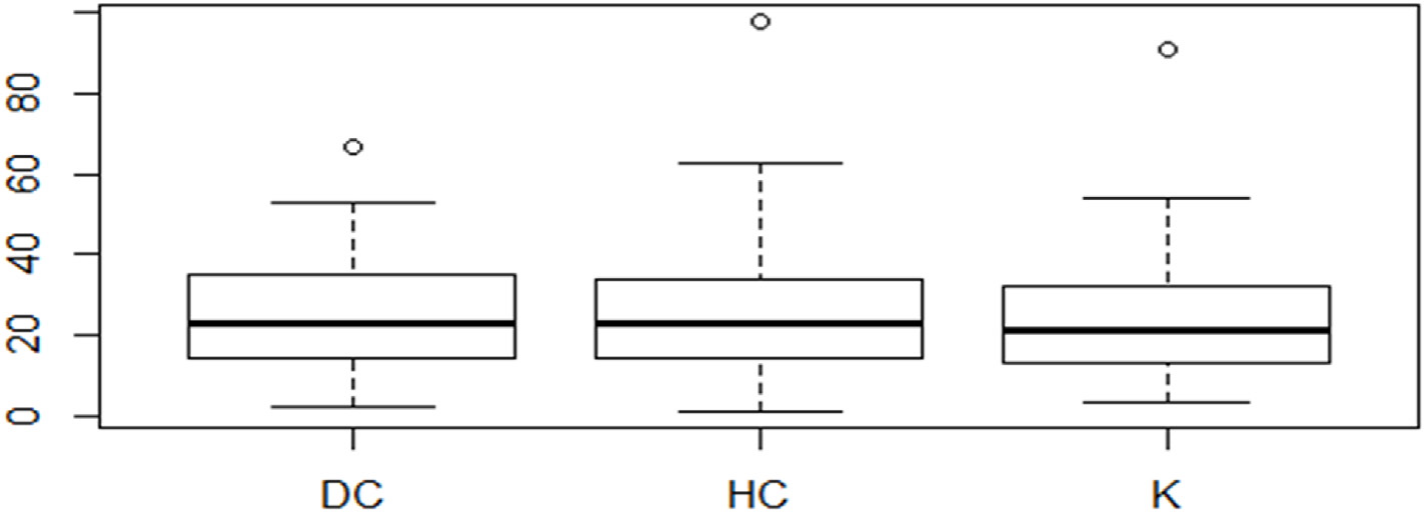
Duration of hospitalization in patients of all three treated groups (shown in days).

Both therapies resulted in adequate control of ICP. Necessity for re-operation for patients with RIH, duration of mechanical ventilation and length of stay in the NICU were in uniform percentages. The duration of the initial hospitalization was somewhat longer in the HC group, but the total duration of treatment, when the repeated hospitalization due to cranioplasty was included, was significantly larger in the DC group. Chart 1.

Complications which may be directly related to the surgical technique in the DC group were noted in eight patients, while in the HC group it was only two patients. Before and during the research, measurements were clearly defined and noted.

## 5 Statistics

The level of statistical significance defined for the study as the standard p ​< ​0.5 was respected. The statistical software which we utilized in the statistical processing was *program R*. The patient outcomes as measured at the time-point GOS0ms (departure from the intensive care unit) was as follows: in both the DC and HC groups there were 23 patients with satisfactory outcomes, while in group K it was 21. Poor outcomes were found in 20 patients in group DC, in 22 in HC, and in 28 in group K. Using the statistical *program R* we acquired the following test results: X-squared ​= ​1.1664, df ​= ​2, p-value ​= ​0.5581 (Table 2).

**Table 2:** Treatment success in relation to favorable and unfavorable outcome. For a favorable outcome we took the Glasgow Outcome Scale scores: GOS 3, GOS 4 and GOS 5, and for a bad outcome the scores GOS 1 and GOS 2.

Group	Favorable outcome	Unfavorable outcome	Total
**DC**	23	20	43
**HC**	23	22	45
**K**	21	28	49
**Total**	67	70	137

∗DC-decompressive craniectomy; HC-hinge craniotomy, K-control; X-squared ​= ​1.1664, p-value ​= ​0.5581.

The values of the statistical test falls within the confidence interval (CI), meaning the p-value is larger than α, confirming that there is no statistically significant difference between the aforementioned methods, in regards to outcome success. Similarly, a test in regards to positive outcomes, evaluated after 6 months (GOS6ms) confirmed that, in the time period of 6 months following the incident which led to inclusion into the study, the outcome in DC group was 23, in the HC group it was 26, while in the K group 25 patients had favorable outcomes. Conversely, unfavorable outcomes in the DC group were 20, in the HC 19, and in the K group 24 patients. The results of the test were:

X-squared ​= ​0.4382, df ​= ​2, p-value ​= ​0.8032.

There was not a statistically significant difference between methods, meaning the final outcome of patients treated in any manner (according to their group) correlated.

It is also concluded that no statistically significant difference between methods exits when length of hospitalization is in question. Nevertheless, in the subgroup with patients over 50 years of age, a statistically significant difference between methods K and HC group was found. Specifically, the average length of hospitalization belonging to those who underwent the HC method was substantially larger than methods K. This may be explained by the fact that mortality in patients in group K was larger during hospitalization. On the other hand, in group DC there were repeated hospitalizations due to the cranioplasty. The early and late complications are shown in Table 3.

**Table 3:** Complications in patients in our sample, which can lead to a direct connection with decompressive procedures, including those due to cranioplasty, as reported in the literature.

Early complications	DC	HC	Late complications	DC	HC
ICP elevation	l	–	Hydrocephalus	2	l
Ventriculitis/meningitis	2	I	Hemiparesis	l	–
Seizure	l	–	Liquorrhea	l	l
SDH which was operated	2	–	Seizure	–	–
Pneumonia/sinusitis	–	–	SDH which was operated	–	–
Other infections	I	–	Aphasia	–	–
Coagulopathy	–	–	Other disorders	–	–
Mineral abnormalities	–	–	Neuritis	–	–
DVT/PTE	l	l	Infection of the wound	l	–
Bradycardia	–	–	Removing of the cranioplasty	1	–

∗ICP-intracranial pressure; SDH-subdural hematoma; DVT-deep vein thrombosis; PTE-pulmonary thromboembolism; DC-decompressive craniectomy; HC-hinge craniotomy.

Upon analyzing patient samples, method HC has shown to have the lowest probability of worsening from a prior state of relative stability (GOS 3, 4, and 5), meaning the largest likelihood of patient improvement from a poor state (GOS 1 and 2) comparing outcomes in the time periods GOS0ms and GOS6ms.

## 6 Discussion

Drawing upon our own samples of patients, in a controlled prospective study with obtained data, we aimed to identify whether or not the claim that the use of the hinge craniotomy method in treatment of patients with refractory intracranial hypertension exists. Additionally, we aimed to see if this method would provide poor results, in the areas of method success compared to the other two discussed methods. To the best of our knowledge, this study is the first prospective study that formally examined and quantified the impact of two different types of craniectomy - DC and HC, with a different size of bone flap between, and additionally compared them with conservative (medical, conventional) treatment. Finally, this research showed the outcome of patients with RIH who basically had different pathology. Cooper and colleagues (Cooper et al., 2011b) published the results of the DECRA study, a first-class evidence study, which supported decompressive craniectomy in trauma. Hutchinson's group (Hutchinson et al., 2016) reported that craniectomy can be performed as a standard intervention when intracranial pressure remains high (RIH) despite all other measures taken. Aarabi and colleagues (Aarabi et al., 2009) published the results of a landmark study with DC, level II, for the control of ICP in 50 patients. They found that DC lowered ICP to less than 20 ​mmHg in 85% of patients, with a mortality of 14 of 50, while 16 patients remained in a pre-existing vegetative state or were severely and permanently disabled. A total of 20 patients or 40%, were marked as having a good outcome. Decompressive craniectomy was associated with a better-than-expected functional outcome in patients who failed conservative treatment. Unilateral decompressive craniectomy (hemicraniectomy) was not allowed by the DECRA study protocol, meanwhile, it was an option in the RESCUEicp study protocol (Hutchinson et al., 2006b; Kolias et al., 2013). Meta-analysis published by Gul et al. concerning the use of decompressive methods for the treatment of malignant MCA insult demonstrated the positive role of these procedures on patient survival (Gul et al., 2018). However, controversy persists regarding the appropriate indication, timing, and technique of surgical decompression for the treatment of malignant intracranial hypertension after ischemic cerebral infarction. Despite the widespread use of hemicraniectomy for the treatment of RIH, there is no doubt that DC is associated with significant morbidity, including the risk of postoperative epileptic seizures, hydrocephalus, infection, and hematoma progression (Yang et al., 2008b; Honeybul and Ho, 2011; Rocque et al., 2018; Mishra et al., 2021). In addition, there is a need for another surgical procedure - cranioplasty. Also, Kenning and colleagues refer to a brief cost analysis of uncomplicated cranioplasty procedures, including surgical costs and hospitalization in a hospital for an average duration of 2.4 days (Kenning et al., 2012).

During our study, we compared the impact of both types of surgical decompression on postoperative ICP and early clinical outcomes. We found that both HC and DC achieved equivalent ICP control, limiting the need for aggressive medical therapy. Duration of mechanical ventilation and treatment in NICU is similar. As confirmed by postoperative ICP control and radiographic data, none of the patients in the HC group experienced postoperative neurological deterioration or progression to brain herniation. By meticulously analyzing the statistics through the obtained data, and through a detailed analysis of the results of the study, we confirmed that no statistically significant difference exists in regards to efficacy, or more specifically patient outcomes, between methods DC, HC, and K. Method DC may be useful as a last resort therapy for patients with post-traumatic intracranial hypertension refractory to conventional treatment. Comparison of both types of surgical decompression showed that both DC and HC achieved equivalent ICP control, limiting the need for prolonged and aggressive medical, mostly pharmacologic, therapy. The principle of monitoring ICP in TBI and altering treatment according to its levels, in the event that one does not take into account neurological status and CT scan, did not show benefits in regards to patient outcomes. On the other hand, ICP monitoring influences better patient outcomes when it is taken as the basis for conducting therapy in severe TBI, but not for other pathomorphological conditions with RIH. Adeleye refers technique similar to the Hinge craniotomy, leaving a bony flap on the temporalis muscle as a hinge. He claims that he got a comparable result to the one with the classic DC, without serious complications, and quite economical (Adeleye, 2016). Reid et al. have published the results of a study regarding the size of the bone flap during decompression. They found that for an area of 70–160 cm2, flap size was an independent factor in reducing ICP, but not for overall neurological outcome (Reid et al., 2018). Perhaps even more controversial is the role of the HC procedure for large space-occupying cerebral infarcts. Many authors have debated whether leaving the bone flap in place of decompression allows enough space for the edematous brain to keep the ICP under control and prevent cerebral herniation, even if the bone flap is “adjusted” on a mobile bag (Goettler and Tucci, 2007; Schmidt et al., 2007; Ko and Segan, 2007; Valença et al., 2012; Adeleye and Azeez, 2011). Table 4.

**Table 4:** List of published series dealing with Hinge craniotomy, published by the end of 2022. In total, there are seven retrospective, two retrospective-prospective and only one prospective study. In total, less than 200 patients underwent HC were published.

Author	Number of patients	Publication	Study	Characteristics
Goettler CE & Tucci KA	3	J Trauma, 2007	retrospective	described HC
Schmidt JH et al.	25	J Neurosurg, 2007	retrospective	
Ko K & Segan S	5	Neurosurgery, 2007	retrospective	
Kenning TJ et al.	20	Neurosurg Focus, 2009	retrospective	comparation with DC
Valença MM et al.	5	J Neurosurg, 2010	retrospective	
Adeleye AO &Azeez AL	4	Surg Neurol Int, 2011	retrospective	TBI
Kano et al.	21	Neurol Med Chir, 2012	retrospective/prospective	comparation with DC
Kenning TJ et al.	28	J Neurosurg, 2012	retrospective	MCA insult
Adeleye AO	40	J Neurol Surg, A 2016	retrospective/prospective	TBI
Mishra T et al.	31	Neurol India, 2021	prospective	comparation with DC, TBI

HC, Hinge craniotomy; DC, decompresive craniectomy; TBI, traumatic brain injury; MCA, middle cerebral artery.

In our study, we found that early surgical intervention, better GCS upon admission, and relatively younger patients with lower Marshall CT scores upon admission showed that, as expected, better outcomes following decompression in patients with RIH stemming from any cause. Due to the relatively poor quality of life of our patients who survived, it should be taken into account which patients were subjected to operations with DC or HC. Additional large, multi-centric studies are necessary to confirm the role of decompression, most prominently for HC in subarachnoid hemorrhage. For patients with diffuse axonal injury, early bifrontal DC does not lead to improvement in the functional sense compared to the results of conservative treatment. The results of our study showed that HC was at least as efficacious as DC in providing postoperative ICP control in patients with ischemic MCA infarction.

The absence of necessity for cranioplasty was the primary advantage of using HC as opposed to DC. Finally, all obtained results correlate with the claim that the approach to patients with IH should be individualized for each specific patient. Younger patients with IH, when treated with any one of the three questioned methods, following statistical analysis, had largely comparable outcomes. On the other hand, patients with RIH who essentially had severe TBI, diffuse axonal injury and aneurysmal SAH are likely to be better treated with DC.

Patients with temporal contusions of the brain, unilateral edema, massive MCA infarction and acute subdural hematoma (ASDH), were shown to have larger benefit from HC interventions. Patients, however, with hemorrhagic strokes, lobar hematomas, edema due to encephalitis, and venous thrombosis, as well as those older than 55 years of age and with lesions of the left hemisphere, if treated conservatively, likely will have the same or better results than those operated on.

For the confirmation of all the claims recently mentioned, further studies are needed.

In any event, it remains unclear if survival with a severe disability may be considered a satisfactory result from the perspective of the patient and their family. One of the main limitations of our study is the design, because we could not randomize the patients in the groups properly all the time of study. Also a limitation of our study is that part of the patients who were treated conservatively for a long time, eventually underwent craniectomy. In this way, it is possible that an important period of time during which brain changes occurred was lost, so the relatively worse outcome in the K group may also be a consequence of the influence of this subgroup of patients. A similar problem was referred to by Hutchinson and colleagues (Hutchinson et al., 2016).

When it comes to cost effectiveness HC is potentially cheaper than the DC, because there is no need for second surgery, cranioplasty. In developed countries a cost of DC is about US$ 20,000, but there is no a formal cost-effectiveness analysis for HC (Layard Horsfall et al., 2020).

The choice of treatment of patients with RIH must be a team effort, accommodated to each patient according to their characteristics and course of disease, and should be revised daily. For patients with RIH, it is customary to expect a difficult and timely battle for each step of GCS. Unfortunately, a poor outcome should be expected, but an effort to attain a positive outcome has to be continuous, and a sense of awareness that no ideal solution exists for these patients should be developed in the jeopardized patient's family, as well as in all health care professionals.

## 7 Conclusion

Study confirmed that, generally, no statistically significant difference exists in regards to efficacy between by DC and HC treated group of patients. Both DC and HC have similar results in ICP control and rates of early and late complications. Hinge craniotomy has better cosmetic effects and gives a possibility to avoid later cranioplasty.

## Funding

This research did not receive any specific grant from funding agencies in the public, commercial, or not-for-profit sectors.

## Disclosure statement

The authors declare that they have no conflict of interest.

## Ethical approval

All procedures performed in studies involving human participants were in accordance with the ethical standards of the institutional research committee and with the 1964 Helsinki declaration and its later amendments or comparable ethical standards. Ethical approval was waived by our local ethics committee in view of the retrospective nature of the study and all the procedures being performed were part of the routine care.

## Informed consent

The research data analysis had no effect on the participants or their medical care, and did not require additional informed consent. Consent was obtained from all the patients/next of the kin for the publication of Figures, as well as for the medical information reported in the relative figure legends.

## Declaration of competing interest

The authors declare that they have no known competing financial interests or personal relationships that could have appeared to influence the work reported in this paper.
